# Complex regulatory effects of gut microbial short-chain fatty acids on immune tolerance and autoimmunity

**DOI:** 10.1038/s41423-023-00987-1

**Published:** 2023-03-01

**Authors:** Chang H. Kim

**Affiliations:** 1 Department of Pathology, University of Michigan School of Medicine, Ann Arbor, MI 48109 USA; 2 Mary H. Weiser Food Allergy Center, Center for Gastrointestinal Research, and Rogel Center for Cancer Research, University of Michigan School of Medicine, Ann Arbor, MI 48109 USA

**Keywords:** Short-chain fatty acids, Immune tolerance, Diabetes mellitus, Rheumatoid arthritis, Lupus, Multiple sclerosis, Autoimmunity, Lymphocytes

## Abstract

Immune tolerance deletes or suppresses autoreactive lymphocytes and is established at multiple levels during the development, activation and effector phases of T and B cells. These mechanisms are cell-intrinsically programmed and critical in preventing autoimmune diseases. We have witnessed the existence of another type of immune tolerance mechanism that is shaped by lifestyle choices, such as diet, microbiome and microbial metabolites. Short-chain fatty acids (SCFAs) are the most abundant microbial metabolites in the colonic lumen and are mainly produced by the microbial fermentation of prebiotics, such as dietary fiber. This review focuses on the preventive and immunomodulatory effects of SCFAs on autoimmunity. The tissue- and disease-specific effects of dietary fiber, SCFAs and SCFA-producing microbes on major types of autoimmune diseases, including type I diabetes, multiple sclerosis, rheumatoid arthritis and lupus, are discussed. Additionally, their key regulatory mechanisms for lymphocyte development, tissue barrier function, host metabolism, immunity, autoantibody production, and inflammatory effector and regulatory lymphocytes are discussed. The shared and differential effects of SCFAs on different types and stages of autoimmune diseases are discussed.

## Introduction

Autoimmune diseases include more than one hundred different chronic and often life-threatening illnesses and affect 3–7% of human populations in developed countries [1]. Autoimmune diseases target cells expressing specific antigens, and the most prevalent diseases include type 1 diabetes mellitus (T1DM), rheumatoid arthritis (RA), multiple sclerosis (MS), lupus, myasthenia gravis, and celiac disease [2]. Autoimmune diseases are caused, in part, by the loss of immune tolerance, which leads to the deletion of autoreactive T and B cells. The selective deletion of developing lymphocytes expressing autoreactive antigen receptors, such as T-cell receptor (TCR) and B-cell receptor (BCR), is the first line of immune tolerance, but some autoreactive lymphocytes appear to escape this mechanism [3, 4]. Luckily, autoreactive T and B cells that escape central tolerance mechanisms are controlled in the periphery by inhibitory signals and regulatory immune cells, so they do not damage tissues. Moreover, autoreactive lymphocytes frequently become regulatory, rather than effector, lymphocytes to contradictorily contribute to immune tolerance [5]. An emerging group of immune tolerance mechanisms that further rein in autoreactive lymphocytes are influenced by our lifestyle choices that affect diet, the microbiome and microbial metabolites in the gut [6–8].

The adult human colon harbors large numbers (10^13^–10^14^) of microbial cells at a fairly high diversity (400–1000 operational taxonomic units); these microbes are dominated by Firmicutes, Actinobacteria, and Bacteroidetes [9–11]. The human gut microbiome changes with age. Actinobacteria is the most abundant microbe after weaning, and the abundance of Proteobacteria is considerably increased in seniors over 70 years of age [12]. Healthy individuals have balanced compositions of gut microbes, but patients with autoimmune diseases often have dysbiosis or altered microbial composition in the colon [13]. Even subtle changes in the microbiome without overt dysbiosis may contribute to autoimmunity in humans, as exemplified by the distinct effects of different lipopolysaccharide-producing microbes on T1DM [14].

**Fig. 1:**
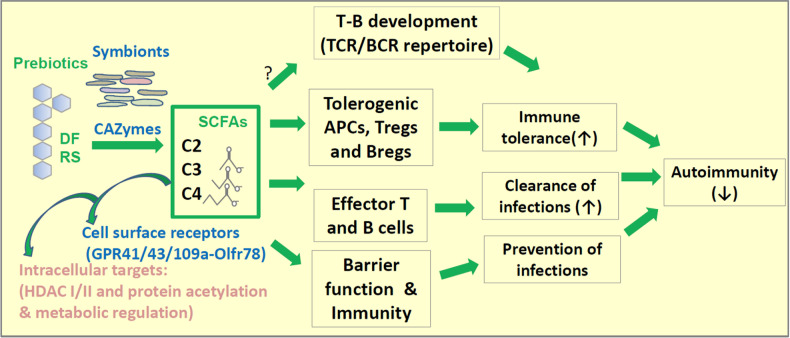
Regulation of immune tolerance by SCFAs. Dietary fiber (DF) and resistant starch (RS) are prebiotics that are processed by microbes to produce SCFAs. Intestinal SCFAs are best produced by certain symbionts expressing CAZymes and/or active SCFA production pathways. By triggering GPR signaling via GPR43, GPR41, GPR109A, and Olfr78, SCFAs affect distinct groups of cells in the body. Intracellular SCFAs, particularly propionate (C3) and butyrate (C4), function as natural HDAC inhibitors. It is expected that certain cell types with high expression of SCFA-transporting solute transporters can effectively concentrate SCFAs within cells for HDAC inhibition. HDAC inhibition triggers elevated gene expression and cell activation to boost tissue barrier functions and the activity of T and B cells. Optimal barrier functions and immune functions are important for preventing infections. HDAC inhibition by SCFAs also generates Tregs, Bregs, tolerogenic antigen presenting cells (APCs such as DCs), and IL-10-producing macrophages, all of which function to suppress inflammatory responses. Microbiota and SCFAs have the potential to shape the antigen receptor repertoire in developing lymphocytes to prevent autoimmunity

In recent years, a plethora of research findings on the functions of the gut microbiota and its metabolites in regulating autoimmune diseases have been reported [15]. Major factors that determine the composition of the gut microbial community include diet and host condition. In general, high microbial diversity with high ratios of protective to pathogenic taxa is beneficial, and these characteristics are promoted by a high prebiotic content in the diet. Aging is a risk factor for autoimmunity that is also linked to disturbances in the balanced gut microbial community [16]. Short-chain fatty acids (SCFAs) are produced from carbohydrates and certain amino acids that reach the colon for bacterial fermentation. SCFAs have important functions in maintaining the gut barrier, host metabolism, immune tolerance and immunity functions (Fig. 1). In autoimmune-associated dysbiosis, the production of SCFAs is frequently altered, which has important ramifications in the immune regulation and pathogenesis of various autoimmune diseases. The functions of SCFAs in supporting immune tolerance and modulating major autoimmune diseases are discussed below.

## Production, receptors and intracellular targets of SCFAs

Major SCFAs, such as acetate (C2), propionate (C3) and butyrate (C4) [17], reach concentrations exceeding 0.1 mol per kg of luminal content in the human colon [18]. They are produced from various prebiotics by fermentation processes such as Wood–Ljungdahl (C2), carbon dioxide fixation (C3), and acetyl-S coenzyme A condensation (C4) processes [18, 19]. Prebiotics include digestion-resistant oligosaccharides (oligofructose), dietary fiber (DF) (e.g., inulin, pectin, and arabinoxylan), and resistant starch (RS) from various plant sources. The structure and composition of DFs vary greatly, depending on the type, content level and plant source [20]. The effects of these prebiotics on SCFA production and gut microbes can differ [20]. Bacterial species that utilize prebiotics express DF-degrading carbohydrate-active enzymes (CAZymes) [21]. Diets rich in DF increase the expression level of microbiome-encoded CAZymes, which have diverse substrate specificity depending on the microbes [22]. Therefore, the level of SCFA production in the colon is largely determined by microbial composition and the types and amount of DF in the diet. Amino acids, excluding branched chain amino acids such as valine, leucine, and isoleucine, can also be metabolized by microbes to produce C2, C3 and C4 [23]. Threonine, for example, can be metabolized to the three major SCFAs. Microbes that are efficient in producing SCFAs are generally considered beneficial and are enriched in the gut of healthy hosts with sufficient levels of DF in their diet [24–26]. In contrast, people consuming low levels of DF or having chronic health conditions, such as autoimmune diseases, are variably deficient in SCFA-producing microbes [27, 28]. In addition to SCFA production and microbial changes, DFs have stool bulking and transit time-shortening effects [29, 30]. Therefore, the effects of DF and SCFAs are not necessarily equal.

SCFAs produced in the gut lumen are absorbed into colonocytes and further transported into the blood circulation by the functions of several solute transporters (SLC5a8, SLC16a1, SLC16a3 and SLC5a12), which are distinctly expressed by different cell types [31]. SCFAs have several different ways to regulate host cells. Some of the functions of SCFAs are mediated through four G-protein-coupled receptors (GPRs): GPR41, GPR43, GPR109A and Olfr78 [32–34]. C4 appears to also activate peroxisome proliferator-activated receptor γ (PPARγ) and Ahr [35–37]. These receptors are selectively expressed by certain cell types in the body. For example, GPR41 is expressed by intestinal epithelial cells, adipocytes, thymic medullary epithelial cells, follicular B cells, germinal center B cells, B1b cells and DCs, whereas GPR43 is expressed by Group 3 innate lymphoid cells (ILC3s), neutrophils, marginal zone B cells, Pre-T cells, DCs, Group 2 innate lymphoid cells (ILC2s), some macrophages and intestinal epithelial cells) [31]. This allows the selective regulation of certain cell types by SCFAs. SCFAs also regulate host cells in several GPR-independent manners. SCFAs that are absorbed into cells can suppress the activity of Class I and II histone deacetylases (HDACs). HDACs deacetylate many proteins, such as histones and signaling molecules, thereby changing their activity [38, 39]. In addition, SCFAs decrease the expression of Type III HDACs, such as sirtuin 1 [36, 37]. Thus, SCFAs increase protein acetylation and therefore affect gene expression and cell signaling. For example, SCFAs increase the acetylation of signaling molecules such as p70 S6 kinase [40]. SCFA-induced acetylation appears to be important for the generation of IL-10-producing B cells [41]. Intracellular SCFAs have metabolic effects in cells. SCFAs are converted into acetyl coenzyme A (acetyl-CoA) to feed into the citric acid cycle for energy production and mTOR activation [40, 42, 43]. Acetyl-CoA that is converted from SCFAs can also increase fatty acid synthesis, which can boost cellular differentiation and functions [42]. The effects of SCFAs on particular cell types appear to be determined by combinations of intracellular functions and cell surface GPR signaling. Of course, different cell types greatly vary in their expression of cell surface GPRs, SCFA transporters, and intracellular targets of SCFAs as a part of cell-specific gene expression programs, leading to diverse and cell type-specific responses to SCFAs.

## Regulation of immune cells and immune tolerance by SCFAs

Developing lymphocytes express antigen receptors randomly selected from recombined DNA sequences in the thymus for T cells and in the bone marrow for B cells. Therefore, they would inherently express autoreactive antigen receptors. Positive and negative selections in the thymus and bone marrow select for lymphocytes with functional receptors that are not autoreactive [44, 45]. Some lymphocytes escape the central selection processes but are regulated by various peripheral tolerance mechanisms [4]. These mechanisms include self-peptide-MHC complex-induced apoptosis, tolerogenic dendritic cells (DCs), and negative costimulatory receptors, such as cytotoxic T-lymphocyte–associated antigen 4 (CTLA-4/CD152) and programmed death-1 (PD-1/CD279). MHC alleles can promote or suppress autoimmune diseases because they control T cells by interacting with TCRs. In this regard, certain MHC alleles affect the gut microbiome and prevent experimental T1DM [46]. The microbiome is important for the development of Treg and non-Treg CD4 T cells and affects their TCR repertoire [47]. Abnormal T-cell development occurs in germ-free mice or pathogenic conditions such as preeclampsia, and this defect was rescued by C2 feeding [48], implying a role for SCFAs in thymic T-cell development. SCFAs and other microbial signals regulate the transition of intestinal DCs to tolerogenic antigen-presenting cells [49]. These DCs, including CX3CR1^+^ DCs, may migrate from the intestine to the thymus to mediate positive selection [50].

**Fig. 2:**
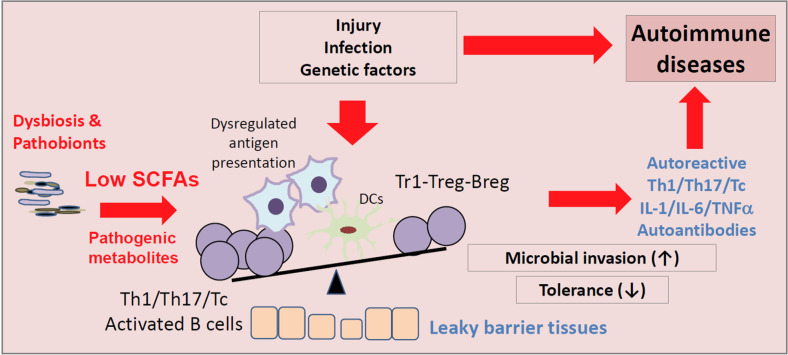
Regulation of immune cells important for immune tolerance by SCFAs. In diseased states following infection or injury, microbial dysbiosis and prolonged inflammatory responses work together to break immune tolerance. Microbial dysbiosis can lead to altered production of microbial metabolites, including decreased SCFAs. SCFA production deficiency may change the activities of APCs, T cells and B cells to generate autoreactive effector lymphocytes. In microbial dysbiosis, pathogenic metabolites, such as certain bile acid derivatives, formate, trimethylamine N-oxide, polyamines, and hydrogen sulfide, are produced instead of SCFAs. These metabolites damage tissues and increase the inflammatory activities of immune cells

SCFAs, particularly C4 and C3, are natural HDAC inhibitors. SCFAs increase IL-10 expression in lymphocytes, and this process appears to be mediated by their HDAC inhibition activity. In this regard, SCFAs promote IL-10^+^ regulatory T-cell activity [51, 52]. SCFAs also promote the production of IL-10 from macrophages. While it is debated whether SCFAs directly induce the generation of FoxP3^+^ T cells from naïve CD4 T cells in the periphery, it is generally accepted that SCFAs enhance the suppressive activity of Tregs [40, 53, 54]. Retinoic acid is a key factor for intestinal immunity and immune tolerance that acts by generating gut-homing effector and regulatory T cells [55–57]. It has also been reported that SCFAs increase the production of retinoic acid from retinaldehyde dehydrogenase (RALDH)-expressing DCs [49, 58]. SCFAs promote tolerogenic macrophages by activating GPRs and inhibiting HDACs [49]. These functions dampen inflammatory responses and suppress autoimmune diseases (Fig. 2).

Certain infections are linked to autoimmune diseases [59]. For example, infection with viruses (rubella, B4 Coxsackievirus, and cytomegalovirus), parasitic protozoa (*Trypanosoma cruzi*), and bacteria (*Borrelia burgdorfii*) are associated with multiple diseases such as T1DM, myocarditis, Chagas disease, multiple sclerosis, and/or Lyme arthritis [60, 61]. Molecular mimicry between pathogens and host antigens and the adjuvant effects of chronic infection are thought to break immune tolerance, leading to autoimmune diseases [61]. Therefore, clearance of pathogens at early stages before they reach chronic states is key to preventing autoimmune diseases. SCFAs are known to boost the functions of Th1 and Th17 cells, which prevent and fight infection [62]. SCFAs increase the production of the effector cytokines IL-22 and IL-17 during active immune responses [63, 64], which are key to preventing infection and fighting pathogens in the intestinal barrier. These functions have the potential to clear infections to prevent chronic inflammatory responses.

B cells can produce autoantibodies, which induce many autoimmune diseases, including RA, systemic lupus erythematosus, autoimmune hepatitis, immune thrombocytopenic purpura, and Guillain‒Barre syndrome [65–67]. SCFAs can serve as nutrients for B cells and enhance B-cell activation during active immune responses [42]. SCFAs are converted into acetyl-CoA, which is used to make long-chain fatty acids needed for plasma B-cell differentiation. SCFAs inhibit HDACs and increase mitochondrial oxidative phosphorylation and glycolysis in B cells, all of which are required for optimal generation of plasma B cells. These functions of SCFAs increase IgG and IgA production to boost humoral immunity. SCFAs increase the numbers of IgA-coated bacteria in the gut, and this is potentially important for the regulation of gut microbes and the prevention dysbiosis [42]. In this regard, SCFAs suppress microbial invasion and leaky gut, which are common in autoimmune diseases [68]. Another function of SCFAs in B cells is to increase the activity of Bregs or B cells that produce IL-10 [41].

The gut is a conducive environment for Tregs that suppress inflammatory responses to microbiota, which may be important for preventing inflammatory and autoimmune responses throughout the body. Important roles of ILCs in promoting intestinal immune tolerance have been reported [69]. It has been reported that MHC II-expressing ILC3s induce Tregs that are selective to microbiota [69]. Another group reported that RORγt^+^ antigen-presenting cells that resemble thymic epithelial cells and/or DCs, but not ILC3s, are required to induce microbiota-specific Tregs in the gut [70, 71]. Independent of their controversial function in inducing Tregs, ILCs produce key effector cytokines of T cells and therefore have the potential to coregulate autoimmune and inflammatory diseases induced by T cells and antibodies. Importantly, SCFAs regulate the activity of ILCs. They increase the proliferation and activity of ILC3s by GPR43 activation but suppress those of ILC2s by HDAC inhibition [31, 72, 73]. These functions of SCFAs are important to maintain barrier immunity and prevent infection and inflammatory diseases.

Leaky barrier functions in the gut, skin and brain are common in autoimmune diseases. These conditions increase infection, tissue damage and antigen exposure, leading to inadvertent activation of the immune system [74–76]. SCFAs maintain the health and barrier function of intestinal epithelial cells. Intestinal epithelial cells highly express SCFA receptors, such as GPR43 and GPR41. Once triggered by SCFAs, GPR43 and GPR41 generate functionally important GPR signaling, leading to mitogen-activated protein kinase kinase (MEK) and extracellular signal-regulated kinase (ERK) activation [77]. GPR activation by SCFAs induces the expression of chemokines, cytokines and antimicrobial peptides, which are necessary for preventing and fighting infection. The HDAC inhibition function of SCFAs enhances the expression of tight junction proteins on intestinal epithelial cells [78, 79]. SCFAs are used by colonocytes as an energy source, supporting the maintenance and growth of intestinal epithelial cells [80]. During microbial invasion, SCFAs facilitate the change in transient permeability in the intestinal barrier to facilitate immune surveillance and activation [77]. SCFAs induce the expression of NLRP3 (NLR Family Pyrin Domain Containing 3) and the inflammasome product IL-18 in a GPR109a- and GPR43-dependent manner [81, 82]. Thus, SCFAs can not only strengthen epithelial barrier function but also boost epithelial innate immune responses to fight pathogens. C4 also acts as a PPARγ ligand and limits the bioavailability of oxygen and nitrate in the colonic lumen. Oxygen and nitrate serve as respiratory electron acceptors, and therefore, this function limits the expansion of potentially pathogenic bacteria in the colonic lumen [83]. Additionally, C4 can induce the expression of tolerogenic TGF-β cytokines in intestinal epithelial cells via HDAC inhibition and transcriptional regulation [84]. Thus, SCFAs have the capacity to promote both epithelial immunity and immune tolerance (Fig. 1).

## Dysbiosis, decreased SCFA production, and leaky gut in T1DM

T1DM is caused by autoimmune destruction of insulin-producing pancreatic islet β-cells. Breakdown of peripheral tolerance to β-cell antigens due to genetic, epigenetic, molecular, and/or environmental issues is thought to cause T1DM [3]. The gut microbiota and SCFAs have the potential to prevent T1DM by promoting immune tolerance through various mechanisms that are described in the previous section. For example, they influence the thymic development of T cells to affect the TCR repertoire and Treg generation [48]. They regulate B cells for effective humoral immune responses and generate Bregs [42, 85]. They also induce tolerogenic antigen-presenting cells [49].

It is generally accepted that microbial composition is altered with decreased levels of the SCFA-producing intestinal microbiome in T1DM. Decreased abundance of Bifidobacterium and Lachnospiraceae and the overabundance of the genera Blautia, Rikenellaceae, Ruminococcus and *Bacteroides ovatus* are observed in T1DM patients [86–88]. In a similar cohort, children who progressed to T1DM had dysbiosis with an altered Bacteroidetes/Firmicutes ratio and SCFA production [89]. Considerable differences in the levels of intestinal SCFAs were detected between T1DM children and the healthy control group, with lower concentrations of fecal C2 and C4 [90]. In this study, an increased abundance of *Ruminococcaceae* and *Coprococcus* but decreased abundance of *Roseburia* and *Megamonas* was observed in the stool samples from children with diabetes. In another study, decreased levels of blood C2 but not C4 in T1DM subjects were observed [91]. Children with a recent-onset of diabetes had gut microbiome dysbiosis and increased intestinal permeability [92]. Subtle microbial changes were also detected in children with anti-islet cell autoantibodies [93].

SCFAs, mainly by their HDAC inhibition functions, protect pancreatic islet cells from inflammatory responses. C4 improved the islet dysfunction induced by oxidative mitochondrial stress [94, 95]. C4 suppressed nuclear factor kappa B (NF-κB) and inflammatory cytokine production by β cells [96]. Moreover, C4 had positive effects on the differentiation of pancreatic islet cells to β cells in vitro and promoted insulin secretion from pancreatic islets [97, 98]. In nonobese diabetic (NOD) mice, long-term antibiotic treatment depleted SCFA-producing bacteria, accelerated disease activity and increased the diabetogenic intestinal microbiome [99]. DF (i.e., inulin) feeding ameliorated streptozotocin-induced diabetes in mice, in part by increasing SCFA production and IL-22 expression [100]. Similarly, an SCFA-producing diet had protective effects in this model with decreased levels of autoreactive T cells and IL-21 but an increased number of Tregs [101]. Protective effects of SCFAs were also observed in NOD mice by suppressing autoimmune T cells but increasing Tregs to suppress pancreatic islet β-cell destruction [90, 101].

Despite the anti-inflammatory and other beneficial effects of SCFAs, β cell loss in advanced T1DM is often permanent, and therefore, it is unlikely that short-term treatment with prebiotics or SCFAs restores β cell functions. This explains why it is difficult to restore β cell function in advanced T1DM with SCFAs and prebiotics. For example, the glucose levels and insulin requirements were unchanged in individuals with diabetes who were treated with SCFA-producing starch (high-amylose maize-RS chemically conjugated with acetate and butyrate) [102]. However, those who had greater changes in SCFA production exhibited elevated levels of glycemic control with increased levels of regulatory T and B cells [102].

**Fig. 3:**
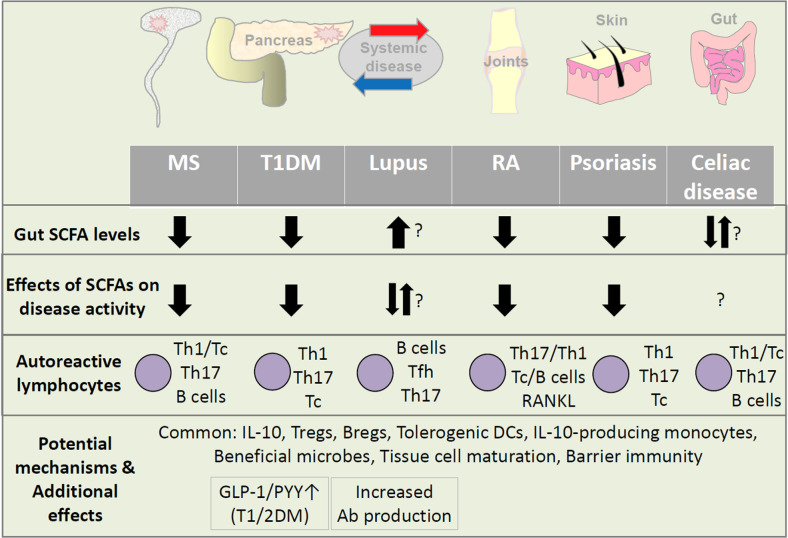
Shared and distinct effects of SCFAs on autoimmune diseases. While there are shared effects of SCFAs on most autoimmune diseases, the effects of SCFAs on the selected autoimmune diseases are heterogeneous. SCFA levels and microbiome diversity are decreased in most autoimmune diseases. Interestingly, SCFA levels are abnormally upregulated in a subset of lupus patients, indicating the presence of disease-specific dysbiosis and/or host metabolism in these subjects. SCFAs are known to activate B cells and induce AID expression to boost class-switched antibody production. Therefore, certain functions of SCFAs have the potential to exacerbate lupus and other autoimmune diseases. Common functions of SCFAs in promoting regulatory lymphocytes, such as Tregs and Bregs, and tolerant antigen-presenting cells appear to operate in most autoimmune diseases. Moreover, microbiota and SCFAs have the potential to shape the antigen receptor repertoire in developing lymphocytes. These functions would prevent autoimmunity in general and decrease ongoing autoimmune responses. In addition, SCFAs have metabolic effects, such as the induction of gut hormone (GLP-1 and PYY) secretion to decrease food intake and inflammatory responses, which could benefit both Type I and II diabetes patients. In contrast, the adverse effect of SCFAs on autoantibody production in lupus and other diseases is a potentially important problem. Despite the progress, it remains still unclear whether SCFAs have significant regulatory effects in many other autoimmune diseases. This is due to either a lack of information or contradictory functions of SCFAs in promoting both the effector and regulatory arms of the immune system

Independent of their immune regulatory functions in T1DM, SCFAs regulate general energy metabolism through various mechanisms. This includes the GPR activation-induced secretion of metabolic hormones, which act on the brain, pancreas, adipocytes, muscle, and liver. GPR43 is highly expressed by enteroendocrine L cells [103, 104]. SCFAs and GPR43 agonists increase the secretion of the gut hormones glucagon-like peptide-1 (GLP-1) and peptide YY (PYY) from L cells [105–107]. In this regard, the effects of SCFAs in suppressing metabolic syndrome and type II diabetes (T2DM) have been well established [108, 109]. These hormones decrease appetite and gastric emptying but increase insulin production, urinary excretion of sodium, and urine production [110]. Additionally, SCFAs increase leptin secretion and insulin sensitivity [111, 112]. Because of these beneficial functions, GLP-1 receptor agonists are used to treat patients with diabetes [113, 114]. While the function of SCFAs in increasing GLP-1 production would metabolically benefit T2DM patients, GLP-1 also has neuroprotective and anti-inflammatory functions [115]. This anti-inflammatory function would also benefit T1DM patients [114]. Thus, SCFAs have the potential to control both T1DM and T2DM by immunological and metabolic regulation (Fig. 3).

## Potentially divergent effects of SCFAs on rheumatoid arthritis and lupus

RA involves chronic inflammation in the joints, leading to destruction of bone and cartilage [116]. RA manifests as symmetric invasive and extra-articular inflammation in multiple joints, and rheumatoid factor and anti-citrullinated protein antibodies are present in the serum of many RA patients. Immune cells and cytokines, such as tumor necrosis factor (TNF), interleukin-1 (IL-1), interleukin-6 (IL-6) and interleukin-18 (IL-18), mediate chronic inflammatory processes [117]. Chronic inflammation increases osteoclastic bone resorption but decreases osteoblastic bone formation for gradual bone and cartilage loss in the joints. Increased Th17 cell responses are frequently involved in RA. These responses lead to the activation of fibroblasts, which increase receptor activator of nuclear factor kappa-B ligand (RANKL) expression and bone resorption [118].

Intestinal and oral dysbiosis in RA patients has been observed with overrepresented intestinal gram-positive bacteria and oral *Porphyromonas gingivalis* and *Lactobacillus salivarius* [119, 120]. It has been reported that levels of stool C3 and C4 were decreased in RA patients [85]. Similar changes in fecal C4 have been observed in animal models, and oral administration of C4 ameliorated experimental RA in mice. This effect may be mediated in part by the function of SCFAs in increasing IL-10-producing B cells. More specifically, the number of AhR^+^ Bregs and the expression of IL-10 by AhR^+^ Bregs were increased by C4. C4 treatment in a mouse model of methylated albumin-induced arthritis decreased inflammation and increased the level of the tryptophan metabolite 5-hydroxyindoleacetic acid (5-HIAA). 5-HIAA triggers the activation of aryl-hydrocarbon receptor (AhR) in B cells, and this increases IL-10 expression [85]. A diet rich in RS had a preventive effect on collagen-induced arthritis (CIA) [121]. RS increased SCFA levels and altered the gut microbial composition. This decreased the abundance of CIA-associated *Lactobacillus* and *Lachnoclostridium* genera but increased the abundance of the *Lachnospiraceae_NK4A136*_group and *Bacteroidales_S24-7*_group genera. Similarly, a high-DF diet increased IL-10 levels but decreased the production of inflammatory cytokines, alleviating disease severity. C3 administration in the drinking water increased Treg cells and IL-10 production [121]. Thus, SCFAs and prebiotics appear to selectively increase the activity of regulatory lymphocytes, such as Tregs and Bregs, over inflammatory effector cells, such as Th17 cells and autoantibody-producing B cells. These immune regulatory effects of prebiotics and SCFAs are consistent with their tolerance maintenance and preventive effects.

In line with the evidence from animal studies, high DF supplementation in RA patients for 4 weeks increased circulating Treg numbers and Th1/Th17 ratios and decreased markers of bone erosion [122]. In another study [123], a high-fiber dietary intervention resulted in a shift in the Firmicutes to Bacteroidetes ratio and increased levels of SCFAs. However, it decreased proarthritic cytokine concentrations. Thus, prebiotics that increase SCFAs are beneficial for RA patients. Prebiotics support the anti-inflammatory effects of SCFAs and increase beneficial microbes that produce other products that also suppress inflammatory responses.

We have witnessed significant levels of controversy regarding the roles of SCFAs in regulating the pathogenesis of lupus. Autoimmune antibodies are the major pathogenic factors for lupus [124]. Low DF intake deteriorated disease progression in lupus-prone NZB/WF1 mice [28]. DF insufficiency exacerbated immune dysregulation and autoantibody production. However, microbiota suppression by antibiotics or direct SCFA feeding did not affect lupus-like disease in this model. DF insufficiency increased fat mass, gut barrier leakage, and low-grade inflammation. A similar beneficial role of RS in suppressing lupus-like pathogenesis in Toll-like receptor 7 (TLR7)-dependent mouse models of systemic lupus erythematosus (SLE) has been reported [125]. Tissue infiltration of *L. reuteri* is associated with the pathogenesis of lupus in this model, and this effect was suppressed by SCFA feeding. This is in line with the effect of DF in preventing dysbiosis and associated inflammatory responses.

DF and SCFAs have strong positive effects on B cells, supporting their activation and differentiation into plasma B cells [42]. This function of SCFAs in promoting humoral immunity could be a potential problem in the pathogenesis of lupus because SCFAs can also increase autoantibody production. In this regard, the levels of C2, C3, and C4 were all increased in fecal samples from lupus patients [126]. A negative association between fecal SCFA levels and the Firmicutes to Bacteroidetes ratio was observed in healthy control individuals, but this was lost in lupus patients. Thus, microbial dysbiosis with increased SCFA production occurs in lupus patients. The positive effect of SCFAs in boosting antibody production is in line with the positive correlation between SCFAs and disease activity in lupus [42].

SCFAs may also regulate the emergence of autoimmune B cells by increasing central and peripheral tolerance mechanisms. Pathogenic autoantibodies in lupus patients are mostly class-switched and hypermutated. Activation-induced deaminase (AID) is required for antibody somatic hypermutation and class switch recombination in B cells, and its expression is regulated by HDAC inhibition [42, 127, 128]. SCFAs are natural HDAC inhibitors and have the potential to regulate AID expression. Indeed, in normal mouse B cells that are activated by BCR activation or during infection by bacterial pathogens, SCFAs increase AID expression potentially through direct epigenetic changes in the *Aicda* gene [42]. Interestingly, SCFAs also increase the expression of several miRNAs that suppress AID expression [129]. Moreover, estrogen, which increases autoimmune antibody production, appears to counteract the SCFA-mediated inhibition of AID expression and CSR [129]. The administration of a high dose of C4 in drinking water modestly suppressed the autoantibody response in autoimmune MRL/Faslpr/lpr mice [129]. While more in-depth studies are needed, the available body of information suggests that autoantibody production may be bidirectionally regulated depending on SCFA levels, B-cell types and the context of immune responses.

## Regulation of MS by microbes and SCFAs

MS pathogenesis involves the demyelination of the central nervous system (CNS), which is caused by chronic immune activation as well as nonimmune mechanisms [130]. There are several types of MS depending on the mode of clinical activity and pathogenesis, including clinically isolated syndrome (CIS), primary progressive MS (PPMS), relapsing-remitting MS (RRMS), and secondary progressive MS (SPMS) [131]. The immune responses mediated by T cells, myeloid cells, and B cells are implicated in MS pathogenesis. Autoimmune T cells target CNS myelin antigens for demyelination. Autoimmune T cells can be activated by viral infection and environmental factors in individuals with certain MHC alleles, such as the human leukocyte antigen (HLA) DRB1*1501 [130]. B cells can bidirectionally regulate MS by presenting autoantigens and producing IL-10 and other immunosuppressive molecules [132]. Microbial dysbiosis occurs in human MS and rodent experimental encephalomyelitis (EAE) models [133, 134]. The abundances of certain microbial species, such as segmented filamentous bacteria (SFB), *P. heparinolytica*, and *S. aureus*, are increased in the gut of hosts with CNS inflammation [135–139]. These microbes increase Th17 activity, which mediates inflammatory responses in the CNS [140, 141]. In contrast, other microbes, such as *Clostridium tyrobutyricum*, which produce SCFAs and other beneficial microbial products, such as polysaccharide A and poly-γ-glutamic acid, are associated with decreased CNS inflammation [142].

The levels of SCFAs in the blood and feces are generally decreased in MS patients [143–147]. MS patients have reduced levels of all major SCFAs (C2, C3, and C4) in the blood [145]. SCFAs increase the number of regulatory T cells and decrease clinical activities in MS patients. C3 ameliorates disease severity in MS patients [148]. However, it has also been reported that C2 was increased in MS patients with severe disability [149, 150]. A caveat is that C2 can be produced by host cells, and the increase may be due to altered host metabolism rather than increased production by gut microbiota [151].

The suppressive effect of SCFAs on experimental CNS inflammation has been observed [152, 153]. This protective effect may be due, in part, to the strengthening effect of SCFAs on the blood brain barrier (BBB) [154–156]. SCFAs also promote microglial cell and oligodendrocyte maturation in a GPR43-dependent manner [155, 156]. Valerate (C5) is another SCFA produced from branched chain amino acids and can increase Tregs and Bregs in a manner potentially dependent on HDAC inhibition [157]. While SCFAs have protective effects, the effect of their precursor, soluble DF, is not entirely clear [158–160]. Soluble DFs, such as pectin and inulin, failed to significantly protect mice from EAE [145]. Highly artificial zero-fiber diets that contained no DF at all, whether soluble or insoluble, exacerbated CNS inflammation compared with a cellulose-containing diet [134, 153]. This finding suggests that most forms of DF, even insoluble cellulose, appear to be beneficial. The beneficial effect of cellulose is likely to be mediated by increased microbial diversity in the gut rather than changes in SCFA production [160]. Surprisingly, the EAE responses of GPR43- or GPR41-deficient mice were lower than those of WT control mice, suggesting that the SCFA effect mediated by their cell surface receptors can even be inflammatory [145]. In human MS patients, elevated levels of DF consumption increased the numbers of Tregs and tolerogenic monocytes in the blood. Coincidentally, the abundance of the Lachnospiraceae phylum also increased. However, a high-DF diet failed to change clinical activity in MS patients [161].

Overall, it is controversial whether SCFAs are effective microbial metabolites that suppress MS pathogenesis. This is not due to the lack of regulatory effects of SCFAs on the CNS immune system. Rather, SCFAs can promote both regulatory and effector responses, and therefore, various outcomes would occur depending on host conditions. What appears clear is that microbial dysbiosis and decreased SCFA production generally occur in MS patients and have the potential to exacerbate ongoing CNS inflammation (Fig. 3).

## Impact on other diseases and closing remarks

The effects of microbially produced SCFAs on immune tolerance and major autoimmune diseases have been discussed in this review. Dysbiosis and inadequate SCFA production have the potential to compromise central and peripheral immune tolerance. The combined information from the four major autoimmune diseases suggests the presence of inflammation-associated dysbiosis and disease-induced lifestyle changes in autoimmune diseases, including inadequate levels of prebiotic consumption. These changes can alter microbial metabolite production, leading to decreased production of SCFAs. However, SCFA production, particularly that of C2, can be upregulated in autoimmune diseases. Consumption of prebiotics at sufficient levels appears beneficial in preventing certain autoimmune diseases. This is mediated by the increased diversity of gut microbes and their metabolites, including SCFAs. SCFAs have profound and complex regulatory effects on diverse types of immune cells. For example, SCFAs can affect host cells via cell surface GPRs, which are differentially expressed by myeloid cells and ILCs. These GPRs are also expressed by metabolically important adipocytes and enterocytes. SCFAs function as natural HDAC inhibitors and promote gene expression in most cell types, leading to balanced activities of immune cells as well as increased tissue integrity. Furthermore, SCFAs serve as nutrients that generate acetyl-CoA and cellular energy. These functions are indeed important to strengthening barrier tissue integrity and preventing autoimmune diseases. It is expected that the functions of SCFAs may vary, depending on the organs that are affected and the type and stage of autoimmune diseases. Because SCFAs support the functions of innate and adaptive immune effector cells, it is likely that certain effects of SCFAs can also exacerbate autoimmune responses during active immune responses [153, 162]. Moreover, late-stage autoimmune diseases are irreversible due to permanent cell loss in affected tissues. While ongoing inflammatory responses may be regulated by SCFA-based approaches, it is unlikely that these approaches can restore lost tissue functions in certain autoimmune diseases, such as T1DM.

Despite the progress thus far in this field, more in-depth studies on the impact of dysbiosis, DFs, and SCFAs on immune tolerance and autoimmune diseases are required. We still do not have enough information regarding the regulatory functions of SCFAs in cells in diverse organs that are targets of autoimmune responses. The study of other autoimmune diseases, including celiac disease for example, is in its infancy. Altered metabolic activity of the intestinal microbial flora in children with celiac disease has been observed [163]. However, there have been mixed reports on SCFA levels in patients with celiac disease. Overall, it is debated whether SCFA levels are significantly altered in celiac disease. Because SCFA levels are altered in many autoimmune diseases and SCFAs can often be anti-inflammatory, the SCFA system composed of SCFAs, their receptors, prebiotics, and SCFA-producing microbes may be targeted to alter the pathogenic course of certain autoimmune diseases. A major caveat is the multifaceted functions of the SCFA system in regulating immune cells and tissue cells, which could make such therapies ineffective and may potentially exacerbate diseases. In this regard, prevention of autoimmune diseases by maintaining optimal microbial diversity and SCFA production by healthy lifestyle choices (e.g., diet, exercise, and sleep cycle) would be more effective.
